# Function of Small Peptides During Male-Female Crosstalk in Plants

**DOI:** 10.3389/fpls.2021.671196

**Published:** 2021-04-23

**Authors:** Jinghua Zhang, Ling Yue, Xiaolin Wu, Hui Liu, Wei Wang

**Affiliations:** College of Life Sciences, National Key Laboratory of Wheat and Maize Crop Science, Henan Agricultural University, Zhengzhou, China

**Keywords:** small peptides, self-incompatibility, pollen germination, polar growth, male-female crosstalk

## Abstract

Plant peptides secreted as signal molecular to trigger cell-to-cell signaling are indispensable for plant growth and development. Successful sexual reproduction in plants requires extensive communication between male and female gametophytes, their gametes, and with the surrounding sporophytic tissues. In the past decade, it has been well-documented that small peptides participate in many important reproductive processes such as self-incompatibility, pollen tube growth, pollen tube guidance, and gamete interaction. Here, we provide a comprehensive overview of the peptides regulating the processes of male-female crosstalk in plant, aiming at systematizing the knowledge on the sexual reproduction, and signaling of plant peptides in future.

## Introduction

Small peptides refer to proteins that are less than 100 amino acids broadly (Hsu and Benfey, 2018), which can be divided into three categories from the source: processed from the original precursor protein; directly translated from an independent small open reading frame (ORF); and encoded by a small ORF in the 5' or 3' untranslated region (UTR) within a normal size protein. Small peptides used as signals are usually secreted proteins which are mainly divided into post-translationally modified small peptides and small cysteine-rich peptides (CRPs; Matsubayashi, 2003). High-throughput sequencing has predicted a large number of small peptide-encoding genes in a variety of plant genomes, and their functions have gradually attracted attention. It is well-established that small peptides are involved in many growth and development processes such as cell proliferation (Imin et al., 2013; Djordjevic et al., 2015), root development (Delay et al., 2013a,b; Mohd-Radzman et al., 2015; Taleski et al., 2016, 2018; Patel et al., 2018), pollen fertility (Okuda et al., 2009; Takeuchi and Higashiyama, 2012, 2016; Higashiyama and Takeuchi, 2015), stomata opening (Takahashi et al., 2018; Qu et al., 2019), absorption and regulation of mineral elements (Taleski et al., 2018), resistance to pests and diseases (Stotz et al., 2009a,b; Ziemann et al., 2018), and environmental adaptation.

**Figure 1:**
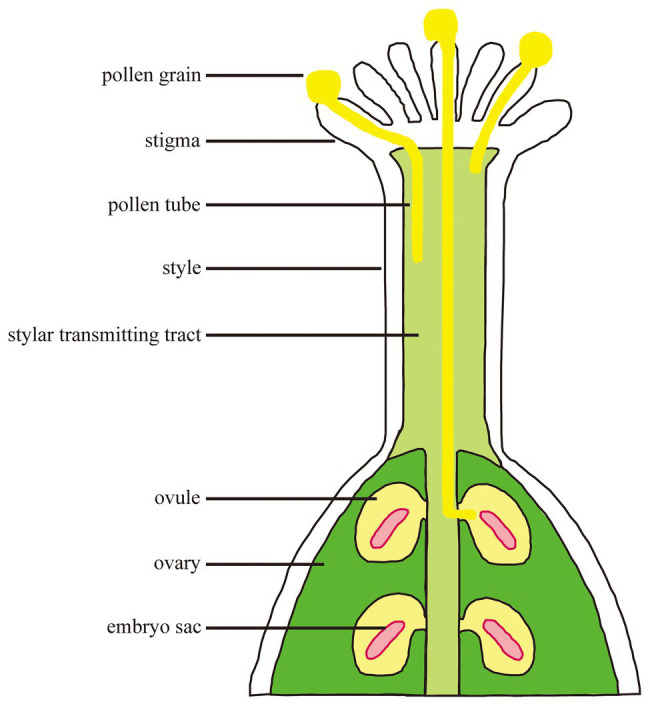
The processes from pollination to fertilization. Pollen grains interact with stigma and germinate. Pollen tube polar growth along the transmitting tract. The pollen tube enters the female gametophyte for fertilization.

Fertilization is a process in which male-female cells interact and fuse with each other in plants. Pollen grains fall onto the stigma through pollination and germinate to form a pollen tube which transports sperm cells through the stigma and grows into the embryo sac along the transmitting tract (Figure 1). A sperm cell fuses with the egg cell to form a zygote which develops into an embryo; the other sperm cell fuses with the central cell to form a fertilized polar nucleus which develops into an endosperm (Hamamura et al., 2012). The successful completion of fertilization relies on the continuous recognition and interaction between female and male cells. The basis for completing these processes is signal communication.

In recent years, a good many of studies have demonstrated the important role of small peptides in male-female crosstalk in plants (Kim et al., 2021). Different small peptides involved in pollen grains-stigma recognition, pollen tube germination, polar growth and reception, ovule attraction, gamete activation, and other processes have been identified. The identification and functional analysis of small peptide during male-female crosstalks are helpful to reveal the formation mechanism of species in plants. Peptide-receptor interaction is the reason for the formation of inter-species isolation. Therefore, researches in this field have great significance for overcoming the reproductive barriers between different species.

## Small Peptides Involved in Important Processes of Plant Reproduction

### Self-Incompatibility

**Figure 2:**
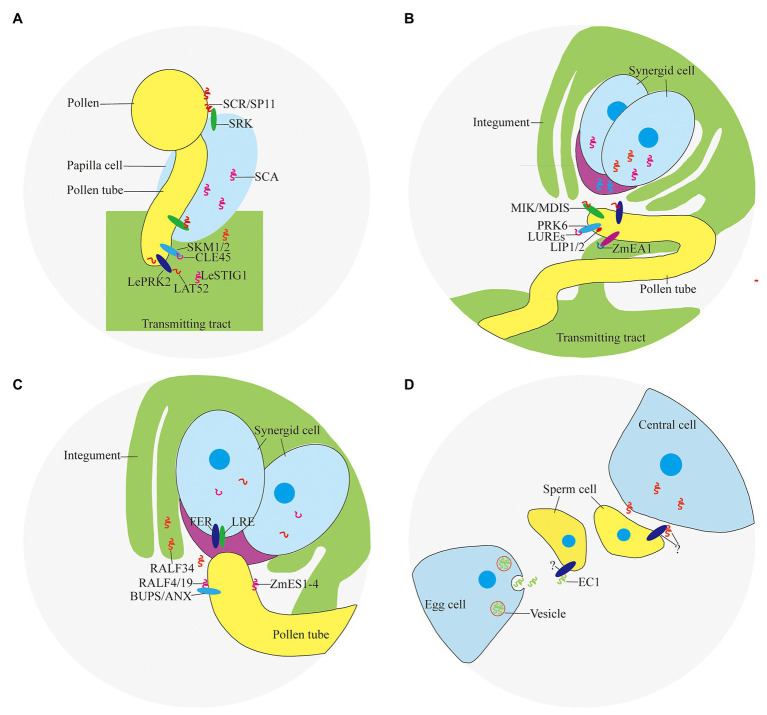
Peptides and receptors involved in double fertilization. **(A)** Pollen-stigma interaction; **(B)** ovule attraction; **(C)** pollen tube reception; and **(D)** gamete activation.

Self-incompatibility is the pre-fertilization reproductive barrier of many plants. The pollen grains fall on the stigma and recognize with the papilla cells quickly before germination, causing interspecific incompatibility and self-incompatibility, preventing different species from crossing and selfing decline (Takayama and Isogai, 2005). Self-incompatibility includes gametophyte self-incompatibility and sporophyte self-incompatibility. The sporophyte self-incompatibility reaction is controlled by the male and female substances encoded by the S locus gene, and the interaction of the S locus encoded protein of the same haplotype inhibits the growth of pollen (tube). Pollen-expressed small peptide ligand S-locus Cys-rich/S-locus protein 11 (SCR/SP11) and small peptide receptor kinase (SRK) on the stigma (Stein et al., 1991; Schopfer et al., 1999; Takayama et al., 2000) play an important role in the determination of sporophyte self-incompatibility in *Brassica napus* (Figure 2A). SCR/SP11 is a small CRP that is secreted into the pollen sac after translation, then transferred and adhered to the surface of the pollen, and interacted with SRK expressed in the stigma papillary cells after being pollinated (Table 1). After SCR/SP11 binds to SRK, the phosphorylation process of multiple factors recruits ubiquitin ligase to degrade the protein Exo70A1 involved in water absorption and hydration, and prevents the germination of pollen tubes (Samuel et al., 2009).

**Table 1:** Peptides and receptors involved in double fertilization.

Biological process	Small peptide	Receptor	References
Self-incompatibility	SCR/SP11	SRK	Schopfer et al., 1999; Takayama et al., 2000
Pollen germination	LAT52	LePRK2	Tang et al., 2002
	PCP-Bα/*β*/*γ*/*δ*	Unknown	Wang et al., 2017
Pollen tube polar growth	LeSTIG1	LePRK2	Tang et al., 2004
	SCA	Unknown	Lord, 2000; Chae et al., 2010
	Plantacyanin	Unknown	Maunoury and Vaucheret, 2011
	Chemocyanin	Unknown	Kim et al., 2006
	CLE45	SKM1/2	Endo et al., 2013
Ovule attraction	LUREs	MDIS1-MIK; LIP1/2	Okuda et al., 2009; Takeuchi and Higashiyama, 2012; Liu et al., 2013; Wang et al., 2016
	ZmEA1	Unknown	Marton et al., 2005, 2012
Pollen tube reception	RALFs	BUPS/ANX	Pearce et al., 2001; Haruta and Constabel, 2003; Haruta et al., 2008; Ge et al., 2017
	ZmES1-4	Unknown	Amien et al., 2010
Gamete activation	EC1	Unknown	Sprunck et al., 2012

### Pollen Germination

The nonmotile sperm cells must rely on the polar growth of the pollen tube to reach the embryo sac. Pollen tube germination and polar growth need the support and guidance of pistil tissue. The signal from the pollen or carpel is received by the receptor on the pollen tube and transmitted to the cell, changing the dynamic nature of the cytoskeleton and forming a pattern of polar growth (Guan et al., 2013).

It was found that PCP-Bα/*β*/*γ*/*δ* located in pollen coat are important for pollen germination because the pollen of *pcp-bα/β/γ/δ* displayed defects in pollen adhesion, pollen hydration, and pollen tube growth *in vivo* (Wang et al., 2017). In tomato, a small CRP LAT52 secreted by pollen is involved in pollen germination (Figure 2A). LAT52 can bind to the pollen tube receptor kinase LePRK2 specifically (Tang et al., 2002). This binding effect is strongest when the pollen tube germinates, and gradually weakens with the extension of the pollen tube. After the pollen tube germinates, substances from the carpel are needed to promote the growth of the pollen tube.

The small CRP LeSTIG1 expressed on stigma of tomato is involved in the regulation of pollen tube growth (Figure 2A). Application of LeSTIG1 can promote pollen tube elongation *in vitro* (Tang et al., 2004). After processing and maturation, LeSTIG1 is secreted out of the cell, combined with the receptor LePRK2, and enriched in the pollen tube. The intracellular domain of LePRK2 interacts with the plant-specific Rop GTPase guanylate exchange factor (GEF) family member KPP, and may regulate pollen tube growth through downstream ROP (Zhang et al., 2008). It has been discovered that the small peptide LeSTIG1 not only binds to receptors, but its C-terminal cysteine-rich domain can also bind to phospholipid molecules such as PI(3)P and participate in intracellular redox state regulation (Huang et al., 2014). Homologous genes of LeSTIG1 in other species may have other functions, such as regulating the secretion of petunia and tobacco stigma cells (Table 1; Verhoeven et al., 2005).

### Pollen Tube Polar Growth

The polar growth of pollen tube is essential for the transportation of sperm cells to embryo sac to complete double fertilization. Small peptides can guide the pollen tube to grow in the transmitting tract of the style. Several CRPs expressed in pistil, such as stigma-style cysteine rich adhesin (SCA; Figure 2A), act as an adhesin binding the pollen tubes to the transmitting tract of the style (Park et al., 2000). The combination of SCA and pectic polysaccharide is necessary to induce pollen tube adhesion to other pollen tubes and to an *in vitro* style matrix (Lord, 2000). SCA is endocytosed into the pollen tube starting at the tip and subsequently moves through an endocytic route. This may be a process triggered by the ligand-receptor binding, but its receptor and the downstream events of the signal have not been clarified (Kim et al., 2006).

In addition, there are gradients formed by plantacyanin and other protein in pistil affect pollen tube elongation. Plantacyanins are secreted proteins with a size of about 10 kDa, with a distinctive gradient from the stigma to the ovule. The distribution may be regulated by the miRNA pathway (Maunoury and Vaucheret, 2011). The pollen tube will elongate at random in the papilla cells and the polar growth will be disrupted if overexpression of the plantacyanin gene to disrupt the gradient distribution pattern. Plantacyanin has properties of copper ion binding, which gives it a higher redox potential, and may participate in the metabolism of reactive oxygen species (ROS). Chemocyanin, the homologous protein of plantacyanin in Lily, is a similar chemotactic factor, which affects the polar growth of pollen tube (Kim et al., 2006).

Flowering plants in the breeding period are particularly susceptible to temperature. CLV3/ESR-related 45 (CLE45), a small modified peptide post-translationally, is involved in the process of maintaining seed yield under high temperature conditions. CLE45 is expressed in the stigma mainly at 22°C, but its expression expands to the transmitting tract upon temperature rise to 30°C. The synthetic CLE domain of CLE45 promotes pollen tube elongation *in vitro* at 30°C. *In vivo*, CLE45 cannot promote elongation, but can prolong the time of pollen tube growth. CLE45 binds the leucine-rich repeat receptor-like kinase STERILITY-REGULATING KINASE MEMBER1 (SKM1) and STERILITY-REGULATING KINASE MEMBER2 (SKM2; Figure 2A). The activity of pollen is maintained through the CLE45-SKM1/SKM2 signaling pathway under high temperature to ensure successful double fertilization (Table 1; Endo et al., 2013).

### Attraction to Ovules

In recent years, it has been confirmed that pollen tubes are attracted by guidance signals from the embryo sac (Palanivelu and Preuss, 2000; Hepler et al., 2001). With the help of laser ablation, Higashiyama et al. observed that a single pollen tube penetrated a synergid cell and discharged its two gametes into the embryo sac as the synergid cell ruptured in *Torenia fournieri*. At the same time, it was found that the effective attracting distance of the synergid cells was 100–200 μm. It implies that the attracting substance has a short diffusible distance and may be secreted small peptides (Higashiyama et al., 2001). LURE1 and LURE2 expressed in the synergid cell abundantly and predominantly are secreted to the surface of the egg apparatus (Figure 2B). LUREs contain six conserved cysteines and are about 65 amino acids in length (~9 KDa). Injection of morpholino antisense oligomers against the LUREs impaired pollen tube attraction, demonstrating that LUREs are the attractants derived from the synergid cells of *T. fournieri* (Okuda et al., 2009).

Studies found that there are more than 300 defensin-like (DEFL) genes involving in cell-to-cell communication during male-female gametes interactions in *Arabidopsis*. AtLURE1 peptides, expressed in egg-accompanying synergid cells specifically, and secreted toward the funicular surface through the micropyle, are pollen tube attractants guiding pollen tubes to the ovular micropyle (Takeuchi and Higashiyama, 2012). In addition, there are still a certain percentage of pollen tubes can be fertilized normally in AtLURE1 RNAi transgenic plants, suggesting that there are other substances involved in pollen tube guidance. *Lost In Pollen tube guidance 1* (*LIP1*) and *2* (*LIP2*) expressed in the membrane of pollen tube, interacted with PRK6 (Figure 2B), perceive the female signal AtLURE1 for micropylar pollen tube guidance (Liu et al., 2013). MALE DISCOVERER1-MDIS1 INTERACTING RECEPTOR LIKE KINASE1 (MDIS1-MIK; Figure 2B), a cell-surface receptor heteromer, was identified to perceive AtLURE1 in *Arabidopsis* (Wang et al., 2016).

In the monocotyledonous maize, *Zea mays* EGG APPARATUS1 (ZmEA1; Figure 2B) expressed in the egg cell and two synergids, is required for pollen tube attraction by the female gametophyte. Transgenic downregulation of the *ZmEA1* gene led to ovule sterility caused by loss of close-range pollen tube guidance to the micropyle (Marton et al., 2005). ZmEA1 is recognized specifically by the pollen tube after being secreted by egg apparatus and degraded subsequently (Table 1; Marton et al., 2012).

### Pollen Tube Reception

The synergid cells not only are required for pollen tube guidance, but also regulate the reception of the pollen tube. The pollen tube enters the female gametophyte by growing into one of the synergid cell which undergoes programmed cell death to burst and release sperm cells (Weterings and Russell, 2004).

The receptor-like serine-threonine kinase FERONIA/SIRENE (FER/SRN) is located on the cell membrane of the synergid cell (Figure 2C). In *feronia* (Huck et al., 2003) and *sirene* (Rotman et al., 2003), pollen tubes of wild-type can enter the embryo sac but fail to cease growth, rupture, and release their contents. Similar pollen tube overgrowths occur in interspecific crosses of *Rhododendron* and in the *in vitro Torenia* system (Higashiyama et al., 1998). It was found that ANXUR1 (ANX1) and ANXUR2 (ANX2; Figure 2C), the pollen-expressed homologs most closely related to FER, function redundantly to control the timing of pollen tube discharge. The pollen tubes of the double-mutant *anx1 anx2* cease growth and burst *in vitro* and fail to reach the embryo sac *in vivo* (Boisson-Dernier et al., 2009; Miyazaki et al., 2009).

Rapid alkalinization factor (RALF), a secreted peptide, suppresses cell elongation of the primary root by activating the cell surface receptor FER in *Arabidopsis* (Haruta et al., 2014). It was found that RALF can induce the signal of Ca^2+^, suggesting an important role in the reception of pollen tubes (Pearce et al., 2001; Haruta and Constabel, 2003; Haruta et al., 2008). BUDDHA’S PAPER SEAL1 and 2 (BUPS1/2) and their peptide ligands RALF4/19 (Figure 2C), are pollen tube-expressed and are required to maintain pollen tube integrity since *ralf4 ralf19* double mutants show pollen tube precocious rupture similar to *anx1 anx2* mutants. Exogenous application of RALF34 peptide induced pollen tube burst (Ge et al., 2017).

*Zea mays* embryo sac (ZmES1-4; Figure 2C), four defensin-like peptides, expressed in synergid cells exclusively, is involved in pollen tube growth arrest, burst, and explosive sperm release. Application of ZmES1-4 results in pollen tube plasma membrane depolarization and sperm cells discharge in maize. The pollen tube-expressed K^+^ channel KZM1 as a target of ZmES1-4, which opens after ZmES1-4 treatment and probably leads to K^+^ influx and sperm release after osmotic burst (Table 1; Amien et al., 2010).

### Gamete Activation

Two sperm cells are released after the pollen tube ruptures. One sperm cell fuses with the egg cell, the other sperm cell fuses with the central cell. This process involved in a large amount of signal transduction, is the last step in establishing species isolation, including cell migration, gamete recognition, cytoplasmic fusion, and nuclear fusion. EGG CELL 1 (EC1; Figure 2D), a small CRP, accumulated in storage vesicles of the egg cell, plays an important role in gamete activation (Table 1). Upon sperm arrival, EC1-containing vesicles are exocytosed. The sperm endomembrane system responds to exogenously applied EC1 peptides by redistributing the potential gamete fusogen HAPLESS 2/GENERATIVE CELL SPECIFIC 1 (HAP2/GCS1) to the cell surface (Sprunck et al., 2012). HAP2/GCS1 located in the inner membrane system of sperm cells is a critical fertilization factor involving in gamete fusion in *Arabidopsis*. Sperm cells of *hap2/gcs1* mutants can be released normally, but cannot fuse with the female gametes, resulting in sterility (Mori et al., 2006; von Besser et al., 2006).

Is there the same mechanism in the central cell? The *Arabidopsis* female gametophytic mutant *glauce* (*glc*) exhibit one sperm cell fuses with the egg cell successfully but the second sperm cell fails to fuse with the central cell, resulting in single fertilization (Leshem et al., 2012). The BAHD transferase involved in secondary metabolism can rescue the fertilization defect of *glc* mutant, implying there may be signals that function similar to EC1 in central cell.

## Conclusion and Perspectives

During the past decade, it has been established that small peptides play an essential role in many developmental processes in plants, such as cell proliferation, maintenance of stem cells, nodule formation, and male-female interaction during plant reproduction (Katsir et al., 2011). Here, we provide a comprehensive overview of the small peptides regulating the processes of male-female crosstalk, including self-incompatibility, pollen tube germination, polar growth and reception, attraction to ovules, and gamete activation (Table 1). Precise interaction between small peptides and the corresponding receptors is essential for reproduction, such as SCR/SP11-SRK regulate self-incompatibility in *B. napus*, LAT52-LePRK2 regulate pollen germination in tomato, CLE45-SKM1/2 regulate pollen tube polar growth, and LUREs-MDIS1/MIK/LIP regulate attraction to ovules.

At present, there are many similar family members of small peptides involved in the process of male-female crosstalk. All or some of them have the same expression pattern, and each of them can bind to the corresponding receptor and function redundantly. However, it is difficult to identify small peptides through traditional genetic and biochemical methods due to the small molecular weight, low content, and high redundancy. More small peptides will be identified with the development of high-throughput sequencing technologies, such as genomics, transcriptomics, and peptidomics.

Small peptides are usually used as ligands and perceived by receptors in male-female communication. The binding of receptor and ligand may cause autophosphorylation of receptor and intracellular phosphorylation cascades. However, many receptors have not been identified yet. It is difficult to identify the corresponding receptors because the ligand is small and the interaction with the receptor is transient. With the great progress of gene editing technology such as CRISPR in recent years, it can be predicted that the research of small peptide will be developed rapidly. In addition, researchers are also interested in how to transfer the signal to the cell after the small peptide and receptor binds.

Fertilization is the premise of seed production. Improving the success rate of fertilization may help to improve crop yield. Understanding the mechanism of male-female crosstalk in the process of reproduction can help solve the problem of self-incompatibility and cross-incompatibility and provide strong support for breeding. The concentration of small peptides to exert their physiological functions is very low. Unlike traditional plant hormones, small peptides are composed of amino acids essentially, and exogenous application will not pose a risk to the environment. The application of small peptides in agricultural production can reduce the amount of pesticides and fertilizers to protect the ecological environment, serving modern green agriculture.

## Author Contributions

JZ and LY wrote the manuscript. XW, HL, and WW provided ideas and revised the manuscript. All authors contributed to the article and approved the submitted version.

### Conflict of Interest

The authors declare that the research was conducted in the absence of any commercial or financial relationships that could be construed as a potential conflict of interest.
